# Generative Bayesian Computation for Maximum Expected Utility

**DOI:** 10.3390/e26121076

**Published:** 2024-12-10

**Authors:** Nick Polson, Fabrizio Ruggeri, Vadim Sokolov

**Affiliations:** 1 Booth School of Business, University of Chicago, Chicago, IL 60637, USA; ngp@chicagobooth.edu; 2 Institute of Applied Mathematics and Information Technologies, Italian National Research Council, 20133 Milan, Italy; fabrizio@mi.imati.cnr.it; 3 Volgenau School of Engineering, George Mason University, 4400 University Drive, MSN 5D3, Fairfax, VA 22030, USA

**Keywords:** generative methods, quantile networks, decision theory, Bayesian computations

## Abstract

Generative Bayesian Computation (GBC) methods are developed to provide an efficient computational solution for maximum expected utility (MEU). We propose a density-free generative method based on quantiles that naturally calculates expected utility as a marginal of posterior quantiles. Our approach uses a deep quantile neural estimator to directly simulate distributional utilities. Generative methods only assume the ability to simulate from the model and parameters and as such are likelihood-free. A large training dataset is generated from parameters, data and a base distribution. Then, a supervised learning problem is solved as a non-parametric regression of generative utilities on outputs and base distribution. We propose the use of deep quantile neural networks. Our method has a number of computational advantages, primarily being density-free and an efficient estimator of expected utility. A link with the dual theory of expected utility and risk taking is also described. To illustrate our methodology, we solve an optimal portfolio allocation problem with Bayesian learning and power utility (also known as the fractional Kelly criterion). Finally, we conclude with directions for future research.

## 1. Introduction

Generative Bayesian Computation (GBC) is a statistical method that estimates the posterior distribution of model parameters when the likelihood function is intractable or hard to calculate. Similar to Approximate Bayesian Computation (ABC), GBC methods are likelihood-free and use simulation methods to estimate the posterior distribution. GBC constructs a probabilistic map to represent a posterior distribution and to calculate functionals of interest. Our goal here is to extend GBC methods to solve maximum expected utility (MEU) problems. We propose a density-free generative method that has the advantage of being able to compute and optimize expected utility as a by-product. To perform this, we find a deep quantile neural map to represent the distributional utility. Then, we provide a key identity which represents the expected utility as a marginal of quantiles.

Although deep learning (DL) has been widely used in business, engineering [1] and finance [2], DL was shown to outperform classical methods for prediction in [3]. Solving optimal decision problems, however, has received less attention. Our work fills this gap and builds on the reinforcement learning literature [4,5], where it is not necessary to know the utilities but, rather, one needs a panel of known rewards and input parameters. The main difference then is our assumption of a utility function [6] and its use in architecture design at the first level of the hierarchy. Recent work on generative methods includes [7,8] in spatial settings, Ref. [9] for causal modeling and [10] for engineering applications.

Our work also builds on [11], whose authors used curve fitting techniques to solve MEU problems. Our work is also related to the reinforcement learning literature of [4]. It differs in that we assume a given utility function, and we directly simulate and model the random utilities implicit in the statistical model. We also focus on density-free generative AI methods. There is a large body of literature on density-based generative methods such as normalized flows or diffusion-based methods. Refs. [12,13] used ABC methods and classification to solve posterior inference problem.

The idea of generative methods is straightforward. Let *Y* denote data and θ a vector of parameters, including any hidden states (also known as latent variables) *z*. Typically, $Y=(y_{1},\ldots ,y_{n})$ is a vector of observations. First, we generate a “look-up” table of “fake” data ${\left\{Y^{\left(i\right)},\theta^{\left(i\right)}\right\}}_{i=1}^{N}$ by simulating a training dataset of parameters and data. This allows us to use deep learning to solve for the inverse map via a supervised learning problem. Generative methods have the advantage of being likelihood-free. For example, our model might be specified by a forward map $Y^{\left(i\right)}=f\left(\theta^{\left(i\right)}\right)$ rather than a traditional random draw from a likelihood function $Y^{\left(i\right)}∼p\left(Y^{\left(i\right)}|\theta^{\left(i\right)}\right)$. Our method works for traditional likelihood-based models but avoids the use of MCMC.

Posterior uncertainty is solved via the inverse non-parametric regression problem, where we predict $\theta^{\left(i\right)}$ from $Y^{\left(i\right)}$. The uncertainty is modeled using $Z^{\left(i\right)}$, which is a random variable that follows some base distribution and is independent of θ. The base distribution is typically uniform, although this does not have to be the case; for example, one could use a very large dimensional Gaussian vector. This approach is justified by the so-called noise outsourcing theorem [14].

**Theorem** **1** (Noise Outsourcing Theorem)**.** *If (Y,Θ) are random variables in a Borel space (Y,Θ), then there exists a r.v. z∼U(0,1), which is independent of Y and a function H:[0,1]×Y→Θ, such that*

$$\left(Y,\Theta \right)\overset{a.s.}{=}\left(Y,H\left(Y,Z\right)\right)$$

Moreover, if there is a statistic S(Y) with Y⊥⊥Θ|S(Y), then

$$\Theta |Y\overset{a.s.}{=}H\left(S\left(Y\right),Z\right).$$

The role of S(Y) is equivalent to the one used in ABC literature. It performs dimension reduction in *n*, the dimensionality of the signal. Our approach is then to first use a deep neural network to calculate the inverse probability map (also known as posterior):

$$\theta \overset{D}{=}F_{\theta |y}^{-1}\left(Z\right),$$

where *Z* is uniform. In the multi-parameter case, we use an RNN or autoregressive structure, where we model a vector via a sequence $\left(F_{\theta_{1}|Y}^{-1}\left(z_{1}\right),F_{\theta_{2}|\theta_{1},Y}^{-1}\left(z_{2}\right)\;\ldots \right)$. A remarkable result from [15] shows that we can learn *S* independent of *H* simply via OLS.

Then, we need to train a deep neural network, *H*, on

$$\theta^{\left(i\right)}=H\left(S\left(Y^{\left(i\right)}\right),Z^{\left(i\right)}\right),$$

Here, S(y) is a statistic to perform dimension reduction with respect to the signal distribution. The existence of *H* follows from the noise outsourcing theorem. Specifying *H* is the key to the efficiency of the approach. Ref. [10] proposes the use of quantile neural networks implemented with ReLU activation functions. Quantile neural network is a deep neural network (DNN) that approximates the quantile function. The training dataset acts as a supervised learning problem and allows us to represent the posterior as a map from input $Y^{\left(i\right)}$ and output $\theta^{\left(i\right)}$. A deep neural network is an interpolator and provides an optimal transport map from base distribution to output. The parameters of the neural network do not need to be identified. The map will provide a probabilistic representation of the posterior for any data vector as we can evaluate the map at any *y*. The question is whether our quantile neural network will generalize well. This is an active area of research and there is a double descent phenomenon that has been found for the generalization risk (see [16,17]).

To extend our generative method to MEU problems, we assume that the utility function *U* is given. Then, we simply draw, from above, the additional associated utilities $U_{d}^{\left(i\right)}:=U\left(d,\theta^{\left(i\right)}\right)$ for a given decision *d* and $\theta^{\left(i\right)}$. Then, we append the utilities to our training dataset, including the baseline distribution $z^{\left(i\right)}$, to yield a new training dataset:

$${\left\{U_{d}^{\left(i\right)},Y^{\left(i\right)},\theta^{\left(i\right)},Z^{\left(i\right)}\right\}}_{i=1}^{N}.$$

Now, we construct a non-parametric estimator of the form

$$U_{d}^{\left(i\right)}=H\left(S\left(Y^{\left(i\right)}\right),\theta^{\left(i\right)},Z^{\left(i\right)},d\right),$$

where *H* is a quantile deep learner and *z* is uniform. Again, S(·) is a summary statistic which allows for dimension reduction in the signal space. A number of authors have discussed the optimal choice of summary statistics, *S*. For example, the authors of [18,19] used deep learning to learn the optimal summary statistics. We add another layer *H* to learn the full posterior distribution map (see also [20,21,22,23,24]).

Given that the posterior quantiles of the distributional utility, denoted by $F_{U|d,y}^{-1}\left(z\right)$, are represented as a quantile neural network, we then use a key identity, which shows how to represent any expectation as a marginal over quantiles, namely

$$E_{\theta |y}\left[U(d,\theta )\right]=\int_{0}^{1}F_{U|d,y}^{-1}\left(z\right)dz$$

This is derived in Section 2.1. The optimal decision function, $d^{★}\left(y\right):=\arg max_{d}E_{\theta |y}\left[U(d,\theta )\right]$, simply maximizes the expected utility. This can then be approximated via Monte Carlo and optimized over any decision variables. We show that quantiles update as composite functions (also known as deep learners) and that the map can be viewed as a concentration function. The Lorenz curve of the utility function can be used to prove the key identity above where expectations are written as marginals of quantiles. There is a similarity with nested sampling [25] and vertical-likelihood Monte Carlo [26].

Our approach focuses on generative density-free quantile methods. Quantile neural networks (QNNs) implemented via deep ReLU networks have good theoretical [27,28] and practical properties. Ref. [29] provides standard non-parametric asymptotic bounds in *N* for the approximation of conditional quantile functions. Ref. [10] proposes the use of quantile posterior representations and the use of ReLU neural networks to perform this task. Rather than dealing directly with densities and the myriad of potential objective functions, we directly model any random variables of interest via a quantile map to a baseline uniform measure. Our neural estimator network directly approximates the posterior CDF and any functions of interest. To solve maximum expected utility problems, we simply add a given utility function as the first layer of the network architecture.

The interpolation property of deep learners is a key feature of our generative AI method as opposed to kernel-based generative methods such as Approximate Bayesian Computation (ABC), which uses accept–reject methods to calculate the posterior at a given output. The authors of [16] pointed out a fascinating empirical property of deep learners in terms of their interpolation approximation properties (see also [17]). There is a second bias–variance trade off in the out-of-sample prediction problem. The interpolation for deep learners suggests that our generative method provides good predictive rules.

Another class of estimators are those based on kernel methods, such as Approximate Bayesian Computations (ABCs). ABC methods differ in the way that they generate their “fake” look-up table. Rather than providing a neural network estimator for any output *y*. ABC methods approximate the likelihood function by locally smoothing using a circle of radius ϵ around the observed data. This can be interpreted as a nearest neighbor model; see [10] for a discussion. The advantage of ABC is that the training dataset is “tilted” towards the observed *y*; the disadvantage is that it uses accept–reject sampling that fails in high-dimensions. Ref. [23] provides theoretical bounds for the generalizability of non-parametric kernel methods.

The rest of this paper is outlined as follows. Section 1.1 first compares GBC methods to ABC posterior simulation. Section 1.1 then provides a description of the generative AI model for learning the utility function. Section 2 defines the distributional utility function and its expectation. Section 3 provides a link with the dual theory of expected utility due to [30]. We introduce the Lorenz curve of the utility function and quantile methods as a way of estimating the posterior expected utility. Section 4 provides an application to portfolio learning. We show how to use generative methods for the normal-normal learning model and to find an optimal portfolio allocation problem based on the Kelly criterion [31]. Section 5 concludes with directions for future research.

### 1.1. Generative Bayesian Computation (GBC)

To fix the notation in the setting of Bayesian parameter inference, let Y denote a locally compact metric space of signals, denoted by *y*, and B(Y) the Borel σ-algebra of Y. Let λ be a measure on the measurable space of signals (Y,B(Y)). Let P(dy|θ) denote the conditional distribution of signals given the parameters. Let Θ denote a locally compact metric space of admissible parameters (also known as hidden states and latent variables z∈Z) and B(Θ) the Borel σ-algebra of Θ. Let μ be a measure on the measurable space of parameters (Θ,B(Θ)). Let Π(dθ|y) denote the conditional distribution of the parameters given the observed signal *y* (also known as the posterior distribution). In many cases, Π is absolutely continuous with density π such that

Π(dθ|y)=π(θ|y)μ(dθ).

Moreover, we will write Π(dθ)=π(θ)μ(dθ) for prior density π when available.

Our framework allows for likelihood and density-free models. In the case of likelihood-free models, the output is simply specified by a map (also known as forward equation):

y=f(θ)

When a likelihood p(y|θ) is available with respect to the measure λ, we write

P(dy|θ)=p(y|θ)λ(dy).

There are a number of advantages of such an approach, primarily the fact that they are density-free. They use simulation methods and deep neural networks to invert the prior to posterior map. We build on this framework and show how to incorporate utilities into the generative procedure.

As a default choice of network architecture, we will use a ReLU network for the posterior quantile map. The first layer of the network is given by the utility function, and hence, this is what makes the method different from learning the posterior and then directly using naive Monte Carlo to estimate the expected utility. This would be inefficient, as quite often, the utility function places high weight on the region of low-posterior probability, representing a tail risk.

Maximum Expected Utility

Decision problems are characterized by a utility function U(θ,d) defined over parameters, θ, and decisions, d∈D. We will find it useful to define the family of utility random variables indexed by decisions as

$$U_{d}:=U\left(\theta ,d\right)\;\;\mathrm{where}\;\;\theta ∼\Pi \left(d\theta \right).$$

Optimal Bayesian decisions [32] are then defined by the solution to the prior expected utility:

$$U\left(d\right)=E_{\theta }\left(U\left(d,\theta \right)\right)=\int U\left(d,\theta \right)p\left(\theta \right)d\theta ,$$

$$d^{★}=\arg\max_{d}U\left(d\right)$$

When information in the form of signals *y* is available, we need to calculate the posterior distribution p(θ|y)=f(y|θ)p(θ)/p(y). Then, we have to solve for the optimal *a posteriori* decision rule $d^{★}\left(y\right)$, defined by

$$d^{★}\left(y\right)=\arg\max_{d}\;\int U\left(\theta ,d\right)p\left(\theta |y\right)d\theta$$

where expectations are now taken with respect to p(θ|y), the posterior distribution.

### 1.2. GBC vs. ABC

GBC works by generating a synthetic dataset to train a quantile neural network, which yields a posterior map (also known as optimal transport) from a base distribution. ABC works by generating a synthetic dataset (the so-called reference table) in the neighborhood of the observed output and uses summary statistics and kernel methods to provide a posterior distribution. As with GBC, this bypasses the need to evaluate a likelihood function or to known densities. Both methods provide a natural alternative to MCMC simulation methods.

Approximate Bayesian Computation (ABC) is a generative method for obtaining samples from the posterior distribution. ABC relies on comparing a summary statistic S(y) to that of the observed output. Denote the training sample by $\left(\theta^{\left(i\right)},Y^{\left(i\right)}\right)$. ABC requires a dimensionality-reducing summary statistic S(y), kernel K(·) and a tolerance level ϵ. The tilted-posterior is defined by

$$\pi_{ABC}^{\epsilon }\left(\theta |Y=y_{obs}\right)=\frac{1}{m_{\epsilon }\left(y_{obs}\right)}\int K_{\epsilon }\left(S\left(y\right)-y_{obs}\right)\lambda_{\theta }\left(dy\right)\pi \left(d\theta \right).$$

Here, $y=(y_{1},\ldots ,y_{n})$ is high-dimensional. Hence, the need for a *k*-dimensional summary statistic $S:{ℜ}^{n}\to {ℜ}^{k}$, where *k* is fixed.

This ensures that the mean of the ABC posterior matches that of the posterior of interest. Furthermore, under a uniform kernel $K=I(|S\left(y\right)-s_{obs}|<eps)$, convergence

$$\pi_{ABC}^{\epsilon }\left(\theta |Y=y_{obs}\right)\to \pi \left(\theta |Y=y_{obs}\right)$$

is guaranteed, namely

$$\begin{array}{cc} \pi_{ABC}^{\epsilon } & =\frac{1}{m_{\epsilon }\left(y_{obs}\right)}\int_{\Theta }I(|S\left(y\right)-s_{obs}|<\epsilon )\delta \left(y-f\left(\theta \right)\right)\pi \left(\theta \right)dyd\theta =\pi (\theta ||S\left(y\right)-s_{obs}\left|<\epsilon \right)\\ \; & \to \pi \left(\theta |Y=y_{obs}\right),\;\mathrm{where}\;S\left(y\right)=\hat{\theta }\left(y\right)=E_{\pi }\left(\theta |y\right). \end{array}$$

An estimator $\hat{\theta }\left(y\right)=S_{\hat{\psi }}\left(y\right)$ can be found using a deep NN, effectively learn a good approximation to the posterior mean from a large dataset ${\left(Y^{\left(i\right)},\theta^{\left(i\right)}\right)}_{i=1}^{N}∼\pi \times M$ and solve the $\ell_{2}$—minimization problem:

$$\arg\min_{\psi }\frac{1}{N}\sum_{i=1}^{N}{∥S_{\psi }\left(Y^{\left(i\right)}\right)-\theta^{\left(i\right)}∥}^{2}.$$

This is equivalent to the high-dimensional non-parametric regression Θ=S(Y)+ϵ and provides methods for estimating the conditional mean. Typically, estimators, $\hat{S}$, include KNN and kernel methods. Recently, deep learners have been proposed and the theoretical properties of superpositions of affine functions (also known as ridge functions) have been provided [23,33].

Generative Bayesian Computation (GBC), on the other hand, takes this approach one step further. Let $Z∼P_{Z}$ be a base measure for a latent variable, *Z*, typically a standard multivariate normal or vector of uniforms. The goal of generative methods is to characterize the posterior measure $P_{\Theta |Y}$ from the training data ${\left(\Theta_{i},Y_{i}\right)}_{i=1}^{N}∼P_{\Theta ,Y}$, where *N* is chosen to be suitably large. A deep learner is used to estimate $\hat{f}$ via the non-parametric regression Θ=H(Y,Z). In this case, *Z* is a base distribution. In the univariate case, where *Z* is uniform, this amounts to inverse cdf sampling, namely $\Theta =F_{\Theta |Y}^{-1}\left(U\right)$.

An important feature of GBC methods is that we have a transport map that holds for *any* output *Y*. We simply evaluate the network at any given *Y*. Moreover, our neural architecture usually includes dimension reduction for the data *Y* via a summary statistic S(Y) and a kernel embedding, typically cosine transform, ψ, for *Z*. Our map is then

Θ=H(S(Y),ψ(Z))

where *Z* is a new base draw. Here, ψ denotes the cosine embedding so that the architecture for the latent variable corresponds to a Fourier approximation with rates of convergence given by $O(N^{-\frac{1}{2}})$ (see Barron (1993)). The deep learner, *H*, is estimated via a quantile NN from the triples ${\left(\Theta_{i},Y_{i},Z_{i}\right)}_{i=1}^{N}∼P_{\Theta ,Y}\times P_{Z}$.

Double Descent

There is still the question of approximation and the interpolation properties of a DNN. Recent research on the interpolation properties of quantile neural networks was recently conducted by [23,27,34]. See also [16,17]. The folklore theorem of deep learning is that shallow deep learners provide good representations of multivariate functions with minimal parameters in the network and are good interpolators.

## 2. Generative Expected Utility

Decision problems under uncertainty are characterized by a utility function U(d,y,θ) defined over decisions, d∈D, signals y∈Y and parameters, θ∈Θ. The *a priori* expected utility is defined by [32] as

$$u\left(d\right)=E_{y,\theta }\left(U\left(d,y,\theta \right)\right)=\int U\left(d,y,\theta \right)d\Pi \left(y,\theta \right).$$

The *a posteriori* expected utility for decision function, d(y), is given by

$$u\left(d,y\right)=E_{\theta |y}\left(U\left(d,y,\theta \right)\right)=\int U\left(d,y,\theta \right)dF_{\theta |y}\left(\theta \right)$$

with expectation taken with respect to posterior cdf.

The distributional form is found by defining the family of utility random variables indexed by decisions defined by

$$U_{d,y}\overset{D}{=}U\left(d,y,\theta \right)\;\;\mathrm{where}\;\;\theta ∼\Pi \left(d\theta |dy\right)$$

Then, we write

$$u\left(d,y\right)=E_{U∼U_{d,y}}\left(U\right)$$

This makes clear the fact that we can view the utility as a random variable defined as a mapping (also known as optimal transport) of (y,θ) evaluated at *d*. Now, we need

$$d^{★}\left(y\right)=\arg\max_{d}u\left(d,y\right).$$

Our deep neural estimator then takes the form

$$U_{d,y}\overset{D}{=}U\left(d,H\left(S\left(y\right),z\right)\right).$$

Generative AI will model θ as a mapping from the data *y* and the quantile *z* as a deep learner. The nonlinear map is then estimated using a simulated training dataset of utilities, signals and parameters, denoted by the set $\{U^{\left(i\right)},Y^{\left(i\right)},\theta^{\left(i\right)}\},\;i=1,\ldots ,N$. We augment this training dataset with a set of independent baseline variables $z^{\left(i\right)},1\leq i\leq N$.

Latent States

We allow for the possibility of further hidden states *z* in the parameter. Our method clearly extends the models that also have hidden states (deterministic or stochastic); for example, many econometric models have deterministic models for the states (e.g., dynamic general stochastic equilibrium (DGSE) models). Our methods are particularly useful for dynamic learning in economics and finance, where MCMC can be computationally prohibitive. We illustrate our method with a portfolio allocation problem and use normal-normal learning. Another class of models where our methods are particularly efficient are the structured sufficient statistics that can depend on hidden latent states and that naturally perform dimensionality reduction for posterior parameter learning (see [35,36]).

### 2.1. Calculating Expected Utility

Expected utility is estimated using a quantile re-ordering trick, and then the optimal decision function maximizes the resulting quantity. We propose using a quantile neural network as the nonlinear map. Notice that we assume that the training data are simulated by the model and are easy to sample, and the simulation costs are low. We can make *N* as large as we want. The key to generative methods is that we directly model the random variable θ as a nonlinear map (deep learner) from the data *y* and the quantile *z*. This is a generalization of the quantile regression to the Bayesian setting.

Quantile Re-ordering

Ref. [4] uses quantile neural networks for decision-making and applies quantile neural networks to the problem of reinforcement learning. Specifically, the authors relied on the fact that expectations are quantile integrals. Let $F_{U}\left(u\right)$ be the CDF of the distributed utility. The key identity follows from the Lorenz curve:

$$E_{U∼U(d,\theta )}\left(U\right)=\int_{0}^{1}F_{U}^{-1}\left(z\right)dz.$$

This key identity follows from the identity

$$\int_{0}^{\infty }udF_{U}\left(u\right)=\int_{0}^{1}F_{U}^{-1}\left(z\right)dz$$

which holds true under the simple transformation $z=F_{U}\left(u\right)$, with Jacobian $dz=f_{U}\left(u\right)du$.

Utility Lorenz Curve

The quantile identity also follows from the Lorenz curve of the utility r.v. as follows. We can compute E(U) using the mean identity for a positive random variable and its CDF or equivalently, via the Lorenz curve. Given the survival function $S_{U}\left(u\right)=1-F_{U}\left(u\right)$, we have

$$\begin{array}{cc} E(U) & =\int_{0}^{\infty }\left(1-F_{U}\left(u\right)\right)du=\int_{0}^{\infty }S_{U}\left(u\right)du\\ E(U) & =\int_{0}^{1}F_{U}^{-1}\left(s\right)ds=\int_{0}^{1}\Lambda \left(1-s\right)ds=\int_{0}^{1}\Lambda \left(s\right)ds \end{array}$$

We do not have to assume that $F_{U}^{-1}\left(s\right)$ or, equivalently Λ(s), is available in closed form, rather we can find an unbiased estimate of this by simulating the Lorenz curve.

The Lorenz curve L of *U* is defined in terms of its CDF, $F_{U}\left(u\right)$, as

$$\begin{array}{cc} L(u) & =\frac{1}{E(U)}\int_{0}^{u}F_{U}^{-1}\left(s\right)ds\;\;\;\mathrm{where}\;\;u\in \left[0,1\right]\\ E_{U∼U(d,\theta )}\left(U\right) & =\int_{\Theta }U\left(d,\theta \right)\Pi \left(d\theta \right)\;. \end{array}$$

One feature of a Lorenz curve is that is provides a way to evaluate

$$E\left(U\right)=\int_{0}^{1}F_{U}^{-1}\left(s\right)ds$$

Hence, we only need to approximate the quantile function $F_{U}^{-1}\left(s\right)$ with a deep Bayes neural estimator.

### 2.2. GenBayes-MEU Algorithm

The method will generalize to the problems of the form

$$\arg\max_{d}u\left(d,y\right)=\int U\left(\theta ,d\right)p\left(\theta |d,y\right)d\theta$$

First, rewrite the expected utility in terms of posterior CDF of a random variable $U_{d}=U\left(d,\theta \right)$, where θ∼p(θ∣d,y) and $U_{d}$ is simply a transformation of $F_{U_{d,y}}^{-1}\left(z\right)$, approximated by a quantile neural network (QNN). We will further approximate the approximate CDF with a quantile neural network. This is a function approximation, which can be achieved using deep learning.

Given the deep learner for the stochastic utilities, namely

$$U_{d}=U\left(H\left(S\left(y\right),z\right),d\right),$$

which is a function of base distribution *z* and the data *y*, we use $y_{obs}$ to draw a value of *U* from *z*. Then, we can use Monte Carlo to estimate the expected utility

$${\hat{U}}^{*}=\frac{1}{N}\sum_{i=1}^{N}U_{d}^{\left(i\right)}.$$

Our algorithm starts by simulating forward ${\left\{y_{i},\theta_{i}\right\}}_{i=1}^{N}$ and proceeds as Algorithm 1:
**Algorithm 1** GBC for MEU  Simulate ${\left(Y^{\left(i\right)},\theta^{\left(i\right)}\right)}_{1\leq i\leq N}∼p\left(y|\theta \right)$ or $Y^{\left(i\right)}=f\left(\theta^{\left(i\right)}\right)$ and $\theta^{\left(i\right)}∼\pi \left(\theta \right)$. 
  Simulate the utility $u^{\left(i\right)}=U\left(d^{\left(i\right)},Y^{\left(i\right)},\theta^{\left(i\right)}\right)$
  Train *H* using the simulated dataset for i=1,…N, via ${\hat{\theta }}^{\left(i\right)}=H\left(Y^{\left(i\right)},z^{\left(i\right)}\right)$  Train *U* using the simulated dataset $U_{d}=U\left(H{\left(S\left(Y^{\left(i\right)}\right),z\right)}^{\left(i\right)},d\right)$ for i=1,…N  Pick a decision *d* that maximizes the expected utility. We use Monte Carlo to estimate the expected utility.
$$E\left(U_{d}\right)=\sum_{i=1}^{N}F_{U_{d}}^{-1}\left(u_{i}\right)\to \underset{d}{\mathrm{maximize}}$$

To find the argmax, we can use several approaches, including Robbins–Monro [37] or temporal differencing (TD) learning [38].

A related problem is that of reinforcement learning and the invariance of the contraction property of the Bellman operator under quantile projections [5].

## 3. Dual Theory of Expected Utility

Similar approaches that rely on the dual theory of expected utility can be found in [30]. How does one evaluate the risky gamble? One way to introduce a utility function on payouts and not change the probabilities and calculate E(u(X)) is to have a distortion measure on the probabilities, also known as the survival function, and leave the payouts alone, and then calculate the expectation of the distorted survival function. A distortion measure is a monotonic function g:[0,1]→[0,1] such that g(0)=0 and g(1)=1. Yaari showed that one can pick distortion *g* to be $u^{-1}$.

Risky prospects are evaluated by a cardinal numerical scale, which resembles an expected utility, except that the roles of payments and probabilities are reversed. Under expected utility, we assess gambles according to

$$E\left(u\left(X\right)\right)=\int_{0}^{\infty }u\left(x\right)p_{X}\left(x\right)dx=\int_{0}^{\infty }u\left(x\right)dF_{X}\left(x\right)$$

The dual theory then will order gambles according to

$$\tilde{E}\left(u\left(X\right)\right)=\int_{0}^{1}g\left(1-F_{X}\left(z\right)\right)dz=\int_{0}^{1}g\left(S_{X}\left(z\right)\right)dz$$

Ref. [30] shows that one can take $g=u^{-1}$ and still obtain the same stochastic ordering of gambles.

$$\int_{0}^{1}u^{-1}\left(1-F_{x}\left(x\right)\right)dx.$$

Specifically, let Y=u(X), and then, picking $g\left(x\right)=S_{x}\left(u^{-1}\left(S_{X}^{-1}\left(x\right)\right)\right)$ yields $S_{Y}\left(t\right)=g\left(S_{X}\left(t\right)\right)$ as required. Hence, the expected utility decomposes as

$$\tilde{E}\left(u\left(X\right)\right)=\int_{0}^{\infty }S_{y}\left(t\right)dt=\int_{0}^{\infty }g\left(S_{x}\left(z\right)\right)dz$$

The function *g*, being monotonic, has similar properties to a distortion function. This provides a class of functions that can be used as a distortion measure. The notion of a concentration function is explored in [39,40,41]. Another key insight is that *g* can be estimated using a deep quantile NN, given a large training sample cam be generated from the direct model.

Distortion (also known as Transformation) Duality

The dual theory has the property that utility is linear in wealth (in the usual framework, the agent would be risk-neutral). To compensate, the agent has to apply a nonlinear transformation known as a distortion measure to the probabilities of payouts. This “tilting” of probabilities is also applied in derivative pricing [42] using a change in measure. In the dual theory, we are interested in the inverses of the distribution function.

The dual theory is motivated by the two representations of the expected value of a random variable, namely

$$E\left(X\right)=\int_{0}^{\infty }\left(1-F_{X}\left(x\right)\right)dx=\int_{0}^{1}F_{X}^{-1}\left(x\right)dx.$$

We will show that the latter is more useful from a computational perspective. Adding risky choice then transforms the inner payouts (standard expected utility) or the probabilities (dual theory).

This also leads to the question of how to calculate and optimize expected utility efficiently using generative methods. We propose the use of a deep neural Bayes estimator.

Let the random utility $U\overset{D}{=}u\left(X\right)$, where $X∼F_{X}$. Let $F_{U}\left(u\right)$ be the corresponding cdf. Then, we can write the expected utility as

$$E\left(U\right)=\int_{0}^{1}udF_{U}\left(u\right)=\int_{0}^{1}\left(1-F_{U}\left(u\right)\right)du=\int_{0}^{1}S_{U}\left(t\right)dt$$

where the de-cumulative distribution (also known as survival) function $S_{U}\left(\cdot \right)$ is defined as

$$S_{U}\left(t\right)=P\left(U>t\right).$$

The survival function is a non-increasing function of *t* and $S_{U}\left(0\right)=1$.

We obtain the dual theory by transforming these survival probabilities. Notice that the dual theory is linear in payouts. The distortion comes from this “risk-neural” probability. Specifically,

$$EU\left(X\right)=\int_{0}^{1}g\left(S_{X}\left(t\right)\right)dt.$$

If *g* is differentiable, then we obtain the so-called Silver formula:

$$EU\left(X\right)=\int_{0}^{1}tg^{\prime }\left(S_{X}\left(t\right)\right)dF_{x}\left(t\right)\;\;\mathrm{with}\;\;\int_{0}^{1}g^{\prime }\left(S_{X}\left(t\right)\right)dF_{X}\left(t\right)=1.$$

Hence, the weights can be interpreted as a tilted probability measure in the dual sense.

If *g* is convex, then $g^{\prime }$ is non-decreasing and

$$EU\left(X\right)=\int_{0}^{1}tg^{\prime }\left(S_{X}\left(t\right)\right)dF_{X}\left(t\right)=\int_{0}^{1}\phi \left(t\right)dF_{X}\left(t\right)=\int_{0}^{1}\phi \left(F_{X}^{-1}\left(z\right)\right)dz.$$

This is a linear utility function and $g^{\prime }\left(S_{X}\left(t\right)\right)$ is a distortion. We can write

$$E\left(U\right)=\int_{0}^{1}\phi \left(t\right)d\left(f\circ F_{X}\right)\left(t\right)=\int_{0}^{1}f\left(F_{X}\left(t\right)\right)d\phi \left(t\right).$$

Appendix A provides a $O(N^{-4})$-error bound for the quantile re-ordering estimator.

## 4. Application

### 4.1. Bayes Rule for Quantiles

Ref. [43] shows that quantile methods are direct alternatives to density computations. Specifically, given $F_{\theta |y}\left(u\right)$, a non-decreasing and continuous from right function, we define

$$Q_{\theta |y}\left(u\right):=F_{\theta |y}^{-1}\left(u\right)=\inf\left(\theta :F_{\theta |y}\left(\theta \right)\geq u\right),$$

which is non-decreasing, continuous from the left. Ref. [43] shows the important probabilistic property of quantiles

$$\theta \overset{P}{=}Q_{\theta }\left(F_{\theta }\left(\theta \right)\right)$$

Hence, we can increase the efficiency by ordering the samples of θ and the baseline distribution and use the monotonicity of the inverse CDF map.

Let g(y) be non-decreasing and continuous from left with $g^{-1}\left(z\right)=\sup\left(y:g(y)\leq z\right)$. Then, the transformed quantile has a compositional nature, namely

$$Q_{g(Y)}\left(u\right)=g\left(Q\left(u\right)\right)$$

Hence, quantiles act as superposition (also known as deep Learner).

This is best illustrated in the Bayesian learning model. We have the following result updating prior to posterior quantiles known as the conditional quantile representation:

$$Q_{\theta |Y=y}\left(u\right)=Q_{\theta }\left(s\right)\;\;\mathrm{where}\;\;s=Q_{F(\theta )|Y=y}\left(u\right)$$

To compute *s* by definition,

$$u=F_{F(\theta )|Y=y}\left(s\right)=P\left(F\left(\theta \right)\leq s|Y=y\right)=P\left(\theta \leq Q_{\theta }\left(s\right)|Y=y\right)=F_{\theta |Y=y}\left(Q_{\theta }\left(s\right)\right).$$

We now provide an application of deep generative quantile networks to Bayesian learning.

### 4.2. Normal-Normal Bayes Learning: Wang Distortion

For the purpose of illustration, we consider the normal-normal learning model. We will develop the necessary quantile theory to show how to calculate posteriors and expected utility without resorting to densities. Also, we show a relationship with Wang’s risk distortion measure as the deep learning that needs to be learned.

Specifically, we observe the data $y=(y_{1},\ldots ,y_{n})$ from the following model:

$$\begin{array}{cc} y_{1},\ldots ,y_{n}|\theta & ∼N(\theta ,\sigma^{2})\\ \theta & ∼N(\mu ,\alpha^{2}) \end{array}$$

Hence, the summary (sufficient) statistic is $S\left(y\right)=\bar{y}=\frac{1}{n}\sum_{i=1}^{n}y_{i}$.

Given the observed samples $y=(y_{1},\ldots ,y_{n})$, the posterior is then $\theta |y∼N(\mu_{*},\sigma_{*}^{2})$ with

$$\mu_{*}=\left(\sigma^{2}\mu +\alpha^{2}s\right)/t,\;\sigma_{*}^{2}=\alpha^{2}\sigma^{2}/t,$$

where

$$t=\sigma^{2}+n\alpha^{2}\;\;\mathrm{and}\;\;s\left(y\right)=\sum_{i=1}^{n}y_{i}.$$

The posterior and prior CDFs are then related via the

$$1-\Phi \left(\theta ,\mu_{*},\sigma_{*}\right)=g\left(1-\Phi \left(\theta ,\mu ,\alpha^{2}\right)\right),$$

where Φ is the normal distribution function. Here, the Wang distortion function is defined by

$$g\left(p\right)=\Phi \left(\lambda_{1}\Phi^{-1}\left(p\right)+\lambda \right),$$

where

$$\lambda_{1}=\frac{\alpha }{\sigma_{*}}\;\;\mathrm{and}\;\;\lambda =\alpha \lambda_{1}\left(s-n\mu \right)/t.$$

The proof is relatively simple and is as follows:

$$\begin{array}{cc} g(1-\Phi \left(\theta ,\mu ,\alpha^{2}\right)) & =g\left(\Phi \left(-\theta ,\mu ,\alpha^{2}\right)\right)=g\left(\Phi \left(-\frac{\theta -\mu }{\alpha }\right)\right)\\ \; & =\Phi \left(\lambda_{1}\left(-\frac{\theta -\mu }{\alpha }\right)+\lambda \right)=1-\Phi \left(\frac{\theta -(\mu +\alpha \lambda /\lambda_{1})}{\alpha /\lambda_{1}}\right) \end{array}$$

Thus, the corresponding posterior updated parameters are

$$\sigma_{*}=\alpha /\lambda_{1},\;\lambda_{1}=\frac{\alpha }{\sigma_{*}}$$

and

$$\mu_{*}=\mu +\alpha \lambda /\lambda_{1},\;\lambda =\frac{\lambda_{1}\left(\mu_{*}-\mu \right)}{\alpha }=\alpha \lambda_{1}\left(s-n\mu \right)/t.$$

We now provide an empirical example.

Numerical Example

Consider the normal-normal model with prior θ∼N(0,5) and likelihood y∼N(3,10). We generate n=100 samples from the likelihood and calculate the posterior distribution.

The posterior distribution calculated from the sample is then θ∣y∼N(3.28,0.98).

**Figure 1:**
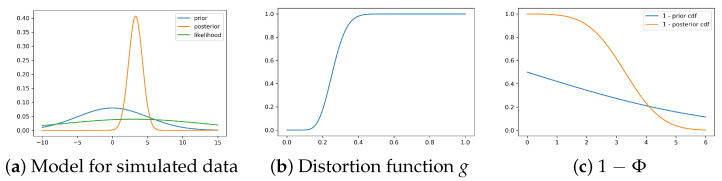
Density for prior, likelihood and posterior, distortion function, and 1 − Φ for the prior and posterior of the normal-normal model.

Figure 1 shows the Wang distortion function for the normal-normal model. The left panel shows the model for the simulated data, while the middle panel shows the distortion function, the right panel shows the 1 − Φ for the prior and posterior of the normal-normal model.

### 4.3. Portfolio Learning

Consider power utility and log-normal returns (without leverage). We assume that a portfolio value $X=e^{W}$ follows a log-normal distribution:

$$W\left(\omega \right)=\left(1-\omega \right)r_{f}+\omega R,\;R∼N\left(\mu ,\sigma^{2}\right)$$

Here, ω∈(0,1) is the portfolio weight, $r_{f}$ is the risk-free rate, μ is the mean return and $\sigma^{2}$ is the variance of the return. The utility function is then given by

$$U\left(W\right)=-e^{-\gamma W}.$$

Here, $U^{-1}$ exists, and the expected utility is

$$U\left(\omega \right)=E\left(-e^{\gamma W}\right)=\exp\left\{\gamma E\left(W\right)+\frac{1}{2}\omega^{2}\mathrm{Var}\left(W\right)\right\}.$$

In this case, we have a closed-form solution for the expected utility, as a function of the decision variable ω (portfolio weight). It is the moment-generating function of the log-normal. We can plug-in the mean and variance of *W* to obtain the expected utility:

$$U\left(\omega \right)=\exp\left\{\gamma \left\{\left(1-\omega \right)r_{f}+\omega \mu \right\}\right\}\exp\left\{\frac{1}{2}\gamma^{2}\omega^{2}\sigma^{2}\right\}.$$

The optimal Kelly–Brieman–Thorpe–Merton value of ω is given by

$$\omega^{*}=\left(\mu -r_{f}\right)/\left(\sigma^{2}\gamma \right).$$

Within the GBC framework, it is easy to add learning or uncertainty on top of $\sigma^{2}$ and have a joint posterior distribution $p(\mu ,\sigma^{2}|R)$.

Now, we re-order the integral in terms of quantiles of the utility function. We assume utility is the random variable and re-order the sum as the expected value of *U*:

$$E\left(U\left(W\right)\right)=\int_{0}^{1}F_{U(W)}^{-1}\left(z\right)dz$$

Hence, if we can approximate the inverse of the CDF of U(W) with a quantile NN, we can approximate the expected utility and optimize over ω.

The stochastic utility is modeled with a deep neural network, and we write

$$Z=U\left(W\right)\approx F,\;W=U^{-1}\left(F\right)$$

We can perform optimization by carrying out the grid search for ω.

The decision variable ω affects the distribution of the returns. The utility only depends on the returns *W*. Our GenAI solution is given by Algorithm 2.
**Algorithm 2** GBC for Portfolio Learning  Simulate log-returns $W^{\left(i\right)}|\omega^{\left(i\right)}∼N\left(\left(1-\omega^{\left(i\right)}\right)r_{f}+\omega^{\left(i\right)}\mu ,\sigma^{2}\omega^{\left(i\right)}2\right)$  Calculate corresponding utilities $Z^{\left(i\right)}=U\left(W^{\left(i\right)}\right)$  Learn $F_{Z_{\omega }}^{-1}$ with a quantile NN  Find the optimal portfolio weight $\omega^{★}$ via
$$E\left(Z_{\omega }\right)=\sum_{i=1}^{N}F_{Z_{\omega }}^{-1}\left(u_{i}\right)\to \underset{\omega }{\mathrm{maximize}}$$

Empirical Example

Consider ω∈(0,1), $r_{f}=0.05$, μ=0.1, σ=0.25, and γ=2. We have the closed-form fractional Kelly criterion solution:

$$\omega^{*}=\frac{1}{\gamma }\frac{\mu -r_{f}}{\sigma^{2}}=\frac{1}{2}\frac{0.1-0.05}{0.25^{2}}=0.40$$

**Figure 2:**
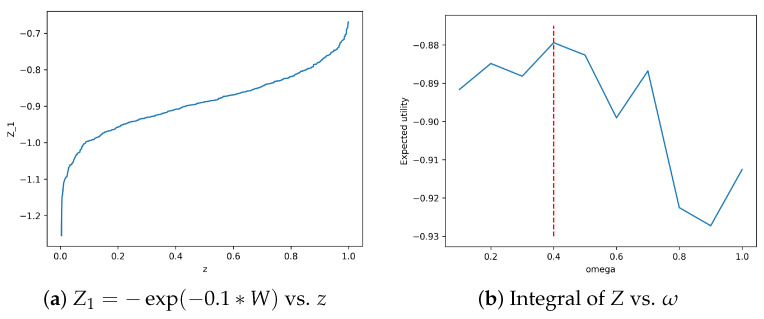
Left panel (**a**) shows plot of sorted values of *z* vs. sorted values of random draws from −exp(−ω*W) for ω=0.1. Right panel (**b**) shows values of integral of *Z* with respect to *z* vs. the corresponding values of ω. The integral was calculated using the trapezoid rule. The red vertical line corresponds to ω=0.4, which is the analytical optimum.

We can simulate the expected utility and compare with the closed-form solution. Figure 2 shows the results of the simulation. The left panel shows the sorted values of the random draws from −exp(−0.1W) vs. the sorted values of the posterior quantiles. The right panel shows the integral of *Z* with respect to ω vs. the corresponding values of ω. The red vertical line corresponds to ω=0.4, which is the analytical optimum.

## 5. Discussion

Generative Bayesian Computation (GBC) is a simulation-based approach to statistical and machine learning. Finding optimal decisions via maximum expected utility is challenging for a number of reasons: first, we need to calculate the posterior distribution over uncertain parameters and hidden states; second, we need to perform integration to find the expected utility; and third, we need to optimize the expected utility. We show how to use deep learning to solve these problems.

We propose a density-free generative method that finds posterior quantiles (and hence, the posterior distribution) via a deep learning estimator. Quantiles are shown to be particularly useful in solving for expected utility densities. Optimization is then performed via a Monte Carlo approximation of the expected utility. We show how to apply this method to the normal-normal model and the portfolio learning problem.

Our goal then was to show their use in solving expected utility problems. It can be viewed as a direct implementation of Yaari’s dual theory of expected utility and to risk distortion measures that are commonplace in risk analysis. There are many avenues for further work; for example, the multi-parameter case and sequential decision problems are two rich areas of future research [44].

Future extensions of this work could include the development of GBC for variable selection, sequential decision-making and general optimization problems. However, we need to note that each application requires a different NN architecture to model the quantiles, and the algorithm may need to be adapted to a specific problem.

## Data Availability

No new data were created or analyzed in this study.

## Appendix A. Calculating Functionals of Interest

Another advantage of quantile methods is that we can find tight error bounds of $O(N^{-4})$ for estimating functionals of interest. Another important property of quantiles is that they can be used to calculate functionals of interest:

$$E_{\theta |y}\left(\theta \right)=\int_{-\infty }^{\infty }\theta \pi \left(\theta |y\right)d\theta =\int_{0}^{1}Q_{\theta |y}\left(z\right)dz$$

We can then use the trapezoidal rule and obtain an efficient approximation due to the lemma of [45].

**Proposition** **A1.**
*Assume Q(x) is a function with a continuous second derivative on [0,1] and*

$$\theta \equiv \int_{0}^{1}Q\left(x\right)dx.$$

*If $Y_{0}\equiv 0,Y_{n+1}\equiv 1$, and ${\left\{Y_{i}\right\}}_{i=1}^{n}$ is the ordered sample associated with n independent uniform observations on [0, 1], then for the estimator*

$$\theta_{n}=\frac{1}{2}\left[\sum_{i=0}^{n}\left(Q\left(Y_{i}\right)+Q\left(Y_{i+1}\right)\right)\left(Y_{i+1}-Y_{i}\right)\right],$$

*we have that for some constant M,*

$$E\left[{\left(\theta_{n}-\theta \right)}^{2}\right]\leq M/n^{4},\;\mathrm{for}\;\mathrm{all}\;n\geq 1.$$

**Proof.** For any particular sample $Y_{1},Y_{2},\ldots ,Y_{n}$, we have

$$\theta -\theta_{n}=\sum_{j=1}^{n}\left(\int_{Y_{j}}^{Y_{j+1}}Q\left(t\right)dt-\frac{1}{2}\left[\left(Q\left(Y_{j}\right)+Q\left(Y_{j+1}\right)\right)\left(Y_{j+1}-Y_{j}\right)\right]\right).$$

A well-known error bound for the trapezoidal rule is that for any number a, b, such that 0≤a<b≤1,

$$\int_{a}^{b}Q\left(t\right)dt-\frac{b-1}{2}\left[Q\left(b\right)+Q\left(a\right)\right]=-\frac{{\left(b-a\right)}^{3}}{12}Q^{\prime \prime }\left(\xi \right),$$

where a≤ξ≤b. Using this result, we have that if $C\geq |Q^{\prime \prime }\left(x\right)|,$0≤x≤1, then

$$\begin{array}{ccc} |\theta -\theta_{n}| & \leq & \left|\int_{0}^{Y_{1}}Q\left(t\right)dt-\frac{1}{2}Y_{1}\left(Q\left(0\right)+Q\left(1\right)\right)\right|\\ & & +\left|\left[\sum_{i=1}^{n-1}\int_{Y_{i}}^{Y_{i+1}}Q\left(t\right)dt-\frac{1}{2}[\left(Q\left(Y_{i}\right)+Q\left(Y_{i+1}\right)\right)\left(Y_{i+1}-Y_{i}\right)\right]\right|\\ & & +\left|\int_{Y_{n}}^{1}Q\left(t\right)dt-\frac{1}{2}[\left(Q\left(Y_{n}\right)+Q\left(Y_{1}\right)\right)\left(1-Y_{n}\right)\right|\\ & \leq & \frac{C}{12}\sum_{i=1}^{n+1}Z_{i}^{3}, \end{array}$$

where $Z_{i}$ are as defined below. Let ${\left\{Y_{i}\right\}}_{i=1}^{n}$ be the ordered sample constructed from *n* independent, uniform observations on the unit interval. Define

$$Z_{1}=Y_{1},\;Z_{j}=Y_{j}-Y_{j-1},\;2\leq j\leq n,\;\mathrm{and}\;Z_{n+1}=1-Y_{n}.$$

Ref. [45] provides the following result: Let ${\left\{Y_{i}\right\}}_{i=1}^{n}$ be the ordered sample constructed from *n* independent, uniform observations on the unit interval, then

$$E\left[Z_{i}^{6}\right]=6!n!/\left(n+6\right)!,\;i=1,2,\ldots ,n+1,$$

$$E\left[Z_{i}^{3}Z_{j}^{3}\right]={\left(3!\right)}^{2}n!/\left(n+1\right)!,\;i,j=1,2,\ldots ,n+1,\;i\neq j.$$

Consequently, letting *i* and *j* range from 1 to n+1, we have

$$E\left[{\left(\theta_{n}-\theta \right)}^{2}\right]\leq E\left[{\left(\frac{C}{12}\sum_{i}Z_{i}^{3}\right)}^{2}\right]=\frac{C^{2}}{144}\left[\sum_{i}E\left[Z_{i}^{6}\right]+\sum_{i\neq j}E\left[Z_{i}^{3}Z_{j}^{3}\right]\right]=O\left(n^{-4}\right).$$

□
